# One-Sided Chronic Intervillositis of Unknown Etiology in Dizygotic Twins: A Description of 3 Cases

**DOI:** 10.3390/ijms22094786

**Published:** 2021-04-30

**Authors:** Lotte E. van der Meeren, Juliette Krop, Kyra L. Dijkstra, Kitty W. M. Bloemenkamp, Emily F. Cornish, Peter G. J. Nikkels, Marie-Louise P. van der Hoorn, Manon Bos

**Affiliations:** 1 Department of Pathology, Leiden University Medical Center, 2333 ZA Leiden, The Netherlands; L.van_der_Meeren@lumc.nl (L.E.v.d.M.); K.L.Dijkstra@lumc.nl (K.L.D.); 2 Department of Pathology, University Medical Center Utrecht, 3584 CX Utrecht, The Netherlands; p.g.j.nikkels@umcutrecht.nl; 3 Department of Immunology, Leiden University Medical Center, 2333 ZA Leiden, The Netherlands; J.Krop@lumc.nl; 4 Department of Obstetrics, Birth Center Wilhelmina’s Children Hospital, University Medical Center Utrecht, 3584 CX Utrecht, The Netherlands; K.W.M.Bloemenkamp@umcutrecht.nl; 5 Elizabeth Garrett Anderson Institute for Women’s Health, University College London, London WC1E 6DB, UK; e.cornish@ucl.ac.uk; 6 Department of Gynecology and Obstetrics, Leiden University Medical Center, 2333 ZA Leiden, The Netherlands; M.L.P.van_der_Hoorn@lumc.nl

**Keywords:** placenta, chronic intervillositis of unknown etiology, twin

## Abstract

Chronic intervillositis of unknown etiology (CIUE) is a rare, poorly understood, histopathological diagnosis of the placenta that is frequently accompanied by adverse pregnancy outcomes including miscarriage, fetal growth restriction, and intrauterine fetal death. CIUE is thought to have an immunologically driven pathophysiology and may be related to human leukocyte antigen mismatches between the mother and the fetus. Dizygotic twins with one-sided CIUE provide an interesting context to study the influence of immunogenetic differences in such cases. The main immune-cell subsets were investigated using immunohistochemistry. We identified three dizygotic twin pregnancies in which CIUE was present in only one of the two placentas. Two of the pregnancies ended in term delivery and one ended in preterm delivery. Presence of CIUE was correlated with lower placental weight and lower birthweight. Relative number of CD68, CD56, CD20, and CD3 positive cells were comparable between co-twins. The presence of one-sided CIUE in dizygotic twin pregnancy was associated with selective growth restriction in the affected twin. This suggests a unique fetal immunogenetic contribution to the pathogenesis of CIUE. Further study of dizygotic and monozygotic placentas affected by CIUE could identify new insights into its pathophysiology and into the field of reproductive immunology.

## 1. Introduction

Chronic intervillositis of unknown etiology (CIUE) is an uncommon histopathological lesion first described by Labarrere and Mullen in 1987 as massive chronic intervillositis [1]. The histiocytic intervillous infiltrate can occur in every trimester and is frequently accompanied by villitis of unknown etiology (VUE) and perivillous fibrin deposits of varying severities [2]. It has repeatedly been shown that CIUE is associated with adverse pregnancy outcomes [3] such as miscarriage, fetal growth restriction (FGR), pre-term birth, and intrauterine fetal death (IUFD) [3].

The pathophysiology of CIUE is poorly understood, but it appears to be immunologically driven [4,5]. Factors that suggest an immune etiology include increased complement deposition in placentas with CIUE [6] and the detection of FOXP3-positive T-cells in the intervillous compartment [7]. CIUE is also associated with maternal auto-immune and alloimmune diseases including antiphospholipid syndrome, systemic lupus erythematosus, and fetal and neonatal alloimmune thrombocytopenia [8,9]. Mixed lymphocyte cultures suggest a pathophysiological basis for human leukocyte antigen mismatches between the mother and the fetus in CIUE [8]. The proposed immune dysregulation in CIUE has led to several experimental treatments with prednisone, corticosteroids, aspirin, heparin, intravenous immunoglobulin, or different in combinations. Adverse outcomes, combined with the condition’s high recurrence rate and the lack of evidence-based treatments for prevention of CIUE, highlight its clinical importance [3].

The phenomenon of dizygotic twins with one-sided CIUE, which occurs despite an identical intrauterine environment, suggests a significant contribution from fetal immunogenetics: the mother mounts an immune response against paternally inherited antigens present in only one of the two placentas. This series describes three cases of dizygotic twins with one-sided CIUE, reporting clinical outcomes, associated placental abnormalities and immunohistochemical findings.

## 2. Results

### 2.1. Patient Characteristics

Clinical characteristics of the dizygotic twin pregnancies are described in Table 1 None of the three affected mothers had any significant past medical history. In case 1, the mother had had two previous pregnancies that both resulted in term delivery of a growth-restricted baby. In Cases 2 and 3, the mothers were primigravidae and in Case 3, became pregnant after ovulation induction. Two of the three pregnancies were delivered at term, and one was delivered pre-term after induction of labor for severe FGR in one of the fetuses. In two of the three dizygotic male-female twin-pregnancies, the fetal growth was discordant. Fetal weight discordance ranged from 9% to 50%. All six babies had an Apgar score above 7 at 5 min, two babies were admitted to the neonatal medium care unit (NMCU), and one baby was admitted to the neonatal intensive care unit (NICU). No IUFD or early neonatal death occurred in these cases. None of the pregnancies was treated (e.g., with prednisone, corticosteroids, aspirin, heparin, intravenous immunoglobulin) and none of the women in our case series had a subsequent pregnancy.

### 2.2. Placental Characteristics

Placental characteristics are described in Table 1. All three placentas were dichorionic diamniotic. The placental weight was within normal range for gestational age for all cases. In each of the three twin pregnancies, CIUE was only present in one of the two placentas. In two cases (1 and 2), CIUE was accompanied by villitis of unknown etiology (VUE). Concurrent fibrin deposits and fetal thrombosis were detected in the CIUE placenta in Case 2.

### 2.3. Quantification of the Intervillous Infiltrate

**Figure 1:**
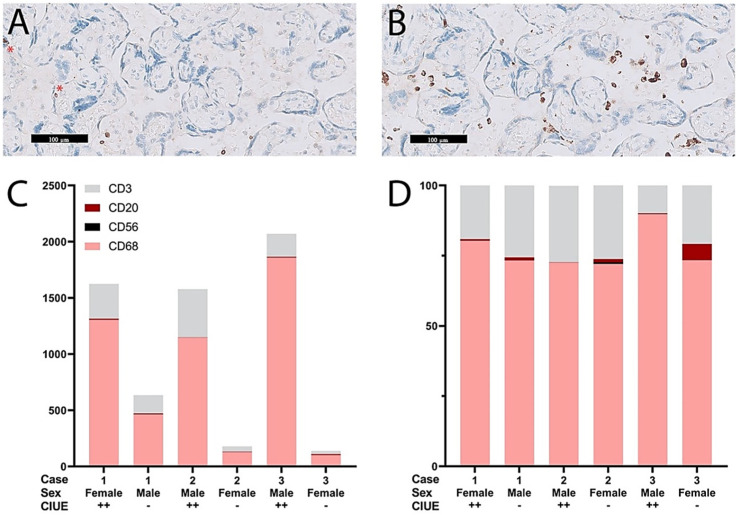
Quantification of the intervillous infiltrate. (**A**) Example of CD3-positive cells in the intervillous space. Two CD3-positive cells are also seen within the villi (see asterisk); (**B**) Example of CD68-positive cells in the intervillous space; (**C**) The absolute number of immune cells in the intervillous space was different among dizygotic twins with and without CIUE; (**D**) The relative number of immune cells in the intervillous space was comparable among co-twins.

The number of cells positive for CD3, CD20, CD56, and CD68 in the intervillous space was quantified (Figure 1A,B). Most of the cells present in the intervillous space were CD68-positive cells, followed by CD3-positive cells. Few CD20-positive cells and CD56-positive cells were observed. The absolute number of cells present in the intervillous space was different between the pairs of co-twin fetuses (Figure 1C), however, the relative number of immune cells present in the intervillous space seems comparable between the pairs of co-twin fetuses (Figure 1D).

### 2.4. Intervillous Cells in Relation to Clinical Outcomes

The fetuses with CIUE in their placenta had a lower birthweight compared to the fetuses without CIUE. In Case 1, the fetus with CIUE was admitted to NMCU; in Case 3, the fetus with CIUE in the placenta was admitted to NICU.

## 3. Discussion

This case series describes three dizygotic twin pregnancies in which the placentas were one-sidedly affected by CIUE, the first published report of this phenomenon. Overall, FGR is more common in twin pregnancies than in singleton pregnancies [10]. Discordant growth is observed in both dichorionic and monochorionic twins, with an incidence of 10–15% [10]. In monochorionic twins, who rely on a single placental circulation, selective FGR is usually driven by unequal placental sharing, in dichorionic twins, FGR has a broader etiology, including genetics, congenital infections, and placental dysfunction [11]. Several histopathological findings in the placenta are associated with FGR such as CIUE, VUE, massive peri-villous fibrin depositions, and fetal thrombosis [12]. One-sided VUE or MPFD in association with FGR have been described in previous studies of dizygotic twins [13,14,15,16,17,18,19]. In our twin cases, the presence of CIUE, with or without peri-villous fibrin deposits or VUE, was also related to a lower birthweight.

One-sided CIUE in the placenta of a dizygotic twin could suggest that CIUE is driven by maternal alloimmune sensitization against a particular fetoplacental histocompatibility antigen which differs between the twins. This is further supported by the presence of CIUE in cases of fetal and neonatal alloimmune thrombocytopenia, which is characterized by maternal allo-antibodies directed against paternally inherited antigens on the fetal platelets [20,21]. The intervillous infiltrate contains predominately CD68-positive cells and CD3-positive cells. The CD68-positve cells represent a non-specific reaction of the maternal immune system. Boyd et al. showed that CD68-positive cells in the intervillous infiltrate express MRP14, which indicates that these cells are activated [22]. The CD3-positive cells resemble a reaction of the adaptive immune system and could detect a non-self-antigen on the fetoplacental cells. This has been confirmed using mixed lymphocyte cultures of peripheral blood mononuclear cells (PBMCs) from women experiencing CIUE and paternal PBMCs as target cells [8]. It could be valuable to ascertain the target antigen of the T-cell receptor on the fetal trophoblast membrane [23,24]. This is particularly interesting in dizygotic twins, since the presented antigens differ between the co-twins. Remarkably, the relative amount of immune cells in the intervillous space of the co-twins is comparable.

One obvious limitation of this series is that it only describes three cases. However, given the rarity of CIUE, which affects approximately 1 in 10,000 pregnancies [25], and the incidence of twin pregnancies, increasing the number of cases is unlikely to overcome the presence of selection bias. As with many studies on placental histopathology, this study is limited by its retrospective design, which also encourages selection bias and makes it more difficult to exclude infectious causes for CIUE. Based on the clinical records, we were able to exclude the presence of clinical signs of infection and histopathological examination of these placentas did not reveal any signs for an infectious cause.

## 4. Materials and Methods

### 4.1. Case Selection

Patient samples were selected from the pathology department of the University Medical Center Utrecht (UMCU) between 2000 and 2015 using the hospital’s pathology registry. During this period, approximately 13,300 placentas were studied, of which 1059 were dichorionic twin placentas. In total, 45 placentas with an intervillous infiltrate were identified, of which three were dizygotic dichorionic twin placentas (see: [26]). Slides were reviewed by experienced pathologists (PGJN and LEvdM) and the diagnosis of CIUE was based on our previously described criteria [3]. We defined CIUE as the presence of an infiltrate occupying 5% or more of the intervillous space with approximately 80% of mononuclear cells positive for CD68 [3]. Furthermore, clinical or histopathological signs of infection should be absent [3]. Patient characteristics and pregnancy outcomes were obtained from the medical records. This study was approved by the UMCU biobank committee (TC-BIO number: 16–434).

### 4.2. Clinical Definitions

Term delivery was defined as birth from 37 completed weeks of gestation onward, and preterm delivery was defined as birth between 24 and 37 completed weeks of gestation. Fetal growth restriction (FGR) was defined as a birthweight below the third percentile. Discordant growth was defined as a birthweight discrepancy of >25%.

### 4.3. Histology and Immunohistochemistry

Tissue samples were taken from the umbilical cord, fetal membranes, and placental parenchyma according to the Amsterdam Placental Consensus Statement [27]. Formalin-fixed, paraffin-embedded (FFPE) tissues were H&E stained according to standard laboratory protocols and studied for routine diagnostics. When CIUE, VUE, or perivillous fibrin deposition was present, it was graded to mild, moderate, and severe/massive based on Benirschke et al. [28]. Fetal thrombosis was scored as either present or absent. For immunohistochemical analysis of the intervillous cell infiltrate in dizygotic twin pregnancies discordant for CIUE, four tissue blocks (two blocks per twin) were selected. Blocks were selected from equivalent locations close to the umbilical cord insertion and halfway between the umbilical cord and lateral edge of the placenta. Blocks from locations with macroscopic abnormalities were excluded. Sequential sections of 4 µm were placed on adhesive-coated glass slides and dried overnight at 37 °C. FFPE tissues were stained for CD3 (rabbit, DAKO A0452; DAKO solutions Ltd., Beverly HU17 0JW, United Kingdom), CD68 (mouse, Monosan NCL-CD-68-KP1; MONOSAN Antibodies and Reagents, Uden, The Netherlands), and CD56 (mouse, Monosan Mon3364; MONOSAN Antibodies and Reagents, Uden, The Netherlands) at the UMCU for routine diagnostics by Ventana (Roche, Tucson, AZ, USA). CD20 staining was performed at the Leiden University Medical Center. Slides were deparaffinized and antigen retrieval was performed with citrate (pH6). Peroxidase was blocked using H_2_O_2_ 0.12%. Prior to incubation with the primary antibody, the slides were incubated for 1 h at room temperature with normal goat serum. The mouse monoclonal anti-CD20 (cy) antibody (1:400, DAKO M0755; DAKO solutions Ltd., Beverly HU17 0JW, United Kingdom) was incubated for 1 h at room temperature. Binding of the primary antibody was visualized using a PO-labelled goat-anti-mouse polymer (DAKO envision; DAKO solutions Ltd., Beverly HU17 0JW, United Kingdom) and diaminobenzidine as a chromogen. Hematoxylin was used for counterstaining before slides were dehydrated and covered using mounting medium.

### 4.4. Quantification of the Intervillous Infiltrate

The number of cells positive for CD3 (T cells), CD20 (B cells), CD56 (NK cells), and CD68 (macrophages) was quantified using the Philips IMS viewer system. The stained slides were aligned and annotations of approximately 0.75 mm^2^ were made per slide. Two annotations were placed subchorial, two were placed central, and two were placed basal (Supplementary Figure S1A). The number of positive cells in the intervillous space was counted for each marker in the aligned annotations (Supplementary Figure S1B–E). In a total of 36 annotations, 5002 CD68-positive cells, 1183 CD3-positive cells, 34 CD20-positive cells, and two CD56-positive cells were counted. All slides were scored with the same brightness settings and magnification. The observer was blinded for clinical outcomes.

## 5. Conclusions

The presence of one-sided CIUE in dizygotic twin pregnancy suggests that CIUE is related to immunological incompatibility between the mother and the affected fetus. Studying one-sided histopathological changes in dizygotic twins and monozygotic twins could reveal novel insights into the pathophysiology of CIUE and other alloimmune gestational diseases.

## Figures and Tables

**Table 1:** Patient characteristics.

Case	1		2		3	
Maternal characteristics						
Gravidity	3		1		1	
Parity	2		0		0	
Outcome previous pregnancy	FGR without complications		NA		NA	
Previous miscarriages	0		NA		NA	
Obstetric characteristics						
Artificial reproductive techniques					Ovulation-induction	
Gestational age (weeks)	37		40		35	
Mode of delivery	Spontaneous		Primary caesarean section		Induction	
Indication for delivery			Twin 1 in breech presentation		Severe fetal growth restriction	
Fetal characteristics						
Sex	Female	Male	Male	Female	Male	Female
Birthweight percentile	p < 3	p10–50	p10–50	p50–90	p < 3	p50–90
Birthweight discordancy	1280 g, 47%		290 g, 9%		1370 g, 50%	
Apgar score at 5 min	8	10	10	10	9	9
Neonatal admission	NMCU				NICU	NMCU
Placenta characteristics						
Fetal Membranes	Dichorionic Diamniotic		Dichorionic Diamniotic		Dichorionic Diamniotic	
Placenta weight percentile	^1^ p10–25		p75–90	p90	p10–25	p75–90
Chronic intervillositis of unknown etiology	Moderate	Absent	Moderate	Absent	Moderate	Absent
Villitis of unknown etiology	Moderate	Absent	Severe	Moderate	Absent	Absent
Peri-villous fibrin deposits	Absent	Absent	Moderate	Absent	Absent	Absent
Fetal thrombosis	Absent	Absent	Present, focal	Absent	Absent	Absent

NICU, neonatal intensive care unit; NMCU, neonatal medium care unit. ^1^ Placenta not weighted separately. Placenta weight percentile for total placenta.

## Data Availability

The data presented in this study are available on request from the corresponding author. Not all the data are publicly available due to privacy considerations.

## Supplementary Materials

The following are available online at https://www.mdpi.com/article/10.3390/ijms22094786/s1, Figure S1: Annotations and alignment.
