# Supplementary material for: One-Sided Chronic Intervillositis of Unknown Etiology in Dizygotic Twins: A Description of 3 Cases
Source: Int J Mol Sci. 2021 Apr 30;22(9):4786. doi: 10.3390/ijms22094786 (PMC8125367; doi:10.3390/ijms22094786)
Supplement: Supplementary file 1 [file ijms-22-04786-s001.zip › ijms-1191263-supplementary.pdf]

Supplementary Materials

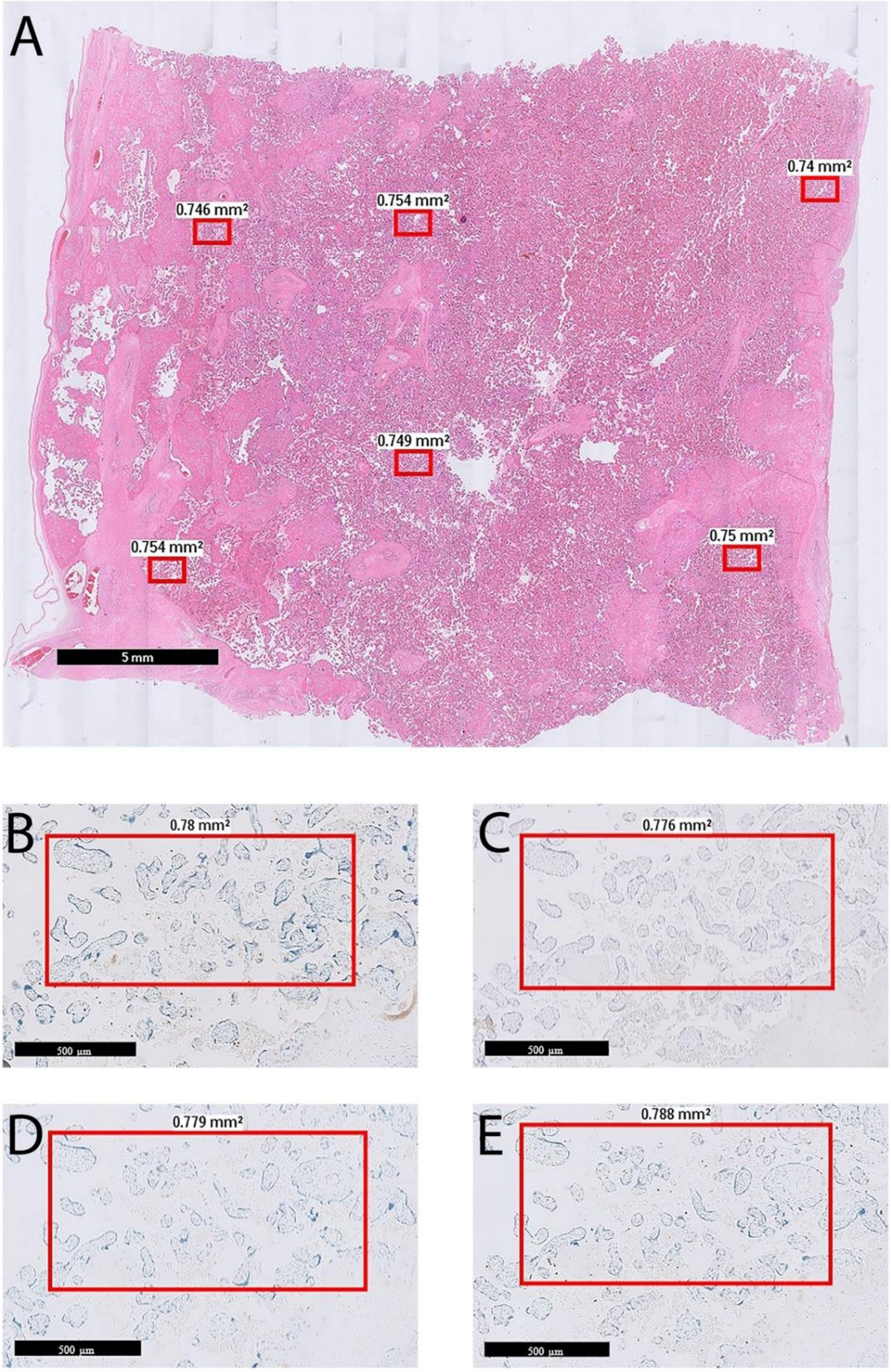

Figure S1. Annotations and alignment. (**a**) An example of selected annotations in a slide stained with H&E. (**b–e**) Examples of aligned annotations per staining: (**b**) CD3; (**c**) CD20; (**d**) CD56; (**e**) CD68.
